# A Differential Signature of Circulating miRNAs and Cytokines Between COVID-19 and Community-Acquired Pneumonia Uncovers Novel Physiopathological Mechanisms of COVID-19

**DOI:** 10.3389/fimmu.2021.815651

**Published:** 2022-01-11

**Authors:** Pedro Martínez-Fleta, Paula Vera-Tomé, María Jiménez-Fernández, Silvia Requena, Emilia Roy-Vallejo, Ancor Sanz-García, Marta Lozano-Prieto, Celia López-Sanz, Alicia Vara, Ángel Lancho-Sánchez, Enrique Martín-Gayo, Cecilia Muñoz-Calleja, Arantzazu Alfranca, Isidoro González-Álvaro, José María Galván-Román, Javier Aspa, Hortensia de la Fuente, Francisco Sánchez-Madrid

**Affiliations:** ^1^ Department of Immunology, Hospital Universitario de La Princesa IIS-IP (Instituto de Investigación Sanitaria del Hospital Universitario de La Princesa), Madrid, Spain; ^2^ Department of Internal Medicine, Hospital Universitario de La Princesa IIS-IP (Instituto de Investigación Sanitaria del Hospital Universitario de La Princesa), Madrid, Spain; ^3^ Data Analysis Unit, Hospital Universitario de La Princesa IIS-IP (Instituto de Investigación Sanitaria del Hospital Universitario de La Princesa), Madrid, Spain; ^4^ Biobank, Hospital Universitario de La Princesa IIS-IP (Instituto de Investigación Sanitaria del Hospital Universitario de La Princesa), Madrid, Spain; ^5^ Department of Medicine, Universidad Autónoma de Madrid (IIS-IP), Madrid, Spain; ^6^ Centro de Investigación Biomédica en Red de Enfermedades Cardiovasculares (CIBERCV), Madrid, Spain; ^7^ Department of Rheumatology, Hospital Universitario de La Princesa IIS-IP (Instituto de Investigación Sanitaria del Hospital Universitario de La Princesa), Madrid, Spain; ^8^ Department of Pneumology, Hospital Universitario de La Princesa IIS-IP (Instituto de Investigación Sanitaria del Hospital Universitario de La Princesa), Madrid, Spain

**Keywords:** COVID-19, community-acquired pneumonia, microRNAs, plasma, soluble proteins

## Abstract

Coronavirus Disease 2019 (COVID-19) pneumonia is a life-threatening infectious disease, especially for elderly patients with multiple comorbidities. Despite enormous efforts to understand its underlying etiopathogenic mechanisms, most of them remain elusive. In this study, we compared differential plasma miRNAs and cytokines profiles between COVID-19 and other community-acquired pneumonias (CAP). A first screening and subsequent validation assays in an independent cohort of patients revealed a signature of 15 dysregulated miRNAs between COVID-19 and CAP patients. Additionally, multivariate analysis displayed a combination of 4 miRNAs (miR-106b-5p, miR-221-3p, miR-25-3p and miR-30a-5p) that significantly discriminated between both pathologies. Search for targets of these miRNAs, combined with plasma protein measurements, identified a differential cytokine signature between COVID-19 and CAP that included EGFR, CXCL12 and IL-10. Significant differences were also detected in plasma levels of CXCL12, IL-17, TIMP-2 and IL-21R between mild and severe COVID-19 patients. These findings provide new insights into the etiopathological mechanisms underlying COVID-19.

## Introduction

Severe Acute Respiratory Syndrome Coronavirus 2 (SARS-CoV-2) is responsible for the disease known as coronavirus disease 2019 (COVID-19) (1). Although initially COVID-19 was defined as a low tract respiratory disease, it is now acknowledged as a complex disorder that compromises multiple organs and may cause long-lasting damage even in patients that overcome the acute phase (2). Major efforts have been devoted to identifying molecules able to predict the severity of COVID-19. These efforts were aimed to improve patient stratification and make a better use of the available resources to optimise health care. After several months of intensive research, some prognostic clinical biomarkers have been identified, including lymphocyte count, D-dimer, C reactive protein (CRP), ferritin, Interleukin (IL)-6 or viremia (3, 4). Moreover, dysregulation of other cytokines, including IL-2, IL-10, Interferon (IFN)-γ, monocyte chemoattractant protein (MCP)-1 or C-X-C Motif Chemokine Ligand (CXCL)10 in COVID-19 patients have also been reported (5).

Some of this research has also been focused on microRNAs (miRNAs), which are short (22 nucleotides in length on average) non-coding RNAs that function as post-transcriptional regulators by binding to mRNAs and preventing protein translation. Each miRNA can target multiple genes, which makes them important regulators of numerous cellular functions (6). Furthermore, miRNAs mediate cell to cell communication travelling either as free circulating molecules or inside exosomes, thereby modulating multiple immune system functions or inflammation in several diseases (7–9). These molecules can be used to predict the clinical course of patients with viral infections, since certain miRNAs are able to bind to distinct RNA viral genomes, blocking their replication (10). A specific miRNA signature has been determined for other viruses, e.g. influenza, which led researchers to find dysregulated pathways in critically ill patients (11).

*In silico* analyses of potentially relevant miRNAs in COVID-19 have been previously carried out. These studies focused on identifying host miRNAs specifically targeting the SARS-CoV-2 genome as a defence strategy (12) or viral miRNAs affecting expression of host genes that could be associated with the pathogenesis of the disease (13). Additionally, previous experimental studies have addressed the relationship of certain miRNAs (miR-2392, miR-146a-5p, miR-21-5p, miR142-3p, miR-15b-5p among others) with the pathogenesis of COVID-19 (14, 15).

However, to our knowledge, no studies have been published that identify miRNAs specifically dysregulated by SARS-CoV-2 infection compared to other types of pneumonia.

The aim of this work was to determine a profile of plasma miRNAs and soluble target molecules in COVID-19 compared to community-acquired pneumonia (CAP) as a specificity control group. CAP was selected because it comprises a group of low tract respiratory infections whose symptoms frequently resemble those of SARS-CoV-2 infection. Here, we identify circulating molecules, such as miRNAs and cytokines, which are differentially expressed in COVID-19 compared to CAP. This study provides novel insights into the molecular mechanisms of this pathology.

## Materials And Methods

### Experimental Design and Patient Selection

To ensure an unbiased manipulation of the samples, a blinded study was carried out. A total of 123 COVID-19 patients were included in this retrospective study. They all had been diagnosed by a positive result of RT-qPCR for SARS-CoV-2 in nasopharyngeal swabs. Patients were admitted to the University Hospital de La Princesa from March 10^th^ to April 21^st^, 2020 (first wave). Plasma samples were collected within 5 days upon admission.

COVID-19 patients were mainly treated with viral protease inhibitors (lopinavir/ritonavir), hydroxychloroquine and/or azithromycin, according to local therapeutic guidelines at the time. Patients treated with either corticosteroids or tocilizumab were excluded from the study. Demographic and clinical variables of the study populations were collected.

The CAP cohort consisted of 33 adult patients, presenting symptoms of lower respiratory tract infection together with the appearance of a new infiltrate on a chest radiograph and the absence of an alternative diagnosis during follow-up, according to the usual definition. All of them were diagnosed with CAP and admitted to the University Hospital de La Princesa between 2014 and 2015. This cohort has been previously used in other studies, in the context of research project on prognostic biomarkers in CAP (16, 17). CAP instead of healthy subjects was selected as the control group because its similarities in symptoms with COVID-19 and to rule out non-specific changes in miRNAs due to a general inflammatory/stress status. Plasma samples were collected at admission, according with ATS/IDSA guidelines in force (18), aliquoted and stored at -80°C until use. These samples were obtained from the Biobank of the University Hospital de La Princesa.

Our screening cohort was composed of 38 COVID-19 patients, 20 with mild disease and 18 with severe disease, classified according to symptoms severity (19). The validation cohort comprised 43 mild and 42 severe COVID-19 patients (**Supplementary Figure 1**).

### Human Plasma Extraction and RNA Purification From COVID-19 Patients

To collect the plasma, EDTA tubes with 10 ml of venous peripheral blood were centrifuged 20 min at 2000 x g at 4°C. 1 ml of plasma was then aliquoted and stored at -80°C for RNA extraction.

Total RNA was isolated from 200 µl of plasma using miRNeasy Serum/Plasma Advanced Kit (QIAGEN), following the manufacturer’s instructions. Plasma samples were centrifuged 5 minutes at 4°C and 3000 x g. 1.25 µg/ml MS2 bacteriophage RNA (Roche Diagnostics) and 1 µl of UniSp2, UniSp4 and UniSp5 RNA spike-in templates were added to each sample as a quality control of the RNA extraction process. The RNA was eluted in 20 µl of RNase-Free Water and then stored at -80°C until use.

To assess hemolysis, absorbance (Abs) at 414 nm of all plasma samples was measured using NanoDrop One/One^C^ Spectrophotometer (Thermofisher Scientific) and those severely hemolysed samples (Abs > 5) were discarded.

### Reverse Transcription (RT) and RT-qPCR Assays

The isolated RNA was reverse transcribed to complementary DNA (cDNA) using miRCURY LNA RT Kit (QIAGEN) following manufacturer’s instructions and quality control of this process was carried out using cel-miR-39 and Unisp6 RNA spike-in templates.

The screening of the candidate miRNAs by qPCR was performed by means of miRCURY LNA miRNA Focus PCR Panels (QIAGEN), which comprises 179 commonly found miRNAs in human plasma.

For qPCR validation assays, miRCURY LNA miRNA Custom PCR Panels (QIAGEN) were used. Both assays were performed using miRCURY LNA SYBR^®^ Green PCR Kit (QIAGEN). QuantStudio 5 Real-Time PCR System (ThermoFisher Scientific) was used for qPCR plates reading.

### qPCR Data Analysis

A quality control of the RNA extraction was performed by means of the spike-in previously added to the extraction and outlier samples were removed from the analysis.

Ct values above 36 were discarded for the global mean calculation. Interplate calibration (IPC) was performed using UniSp3 as described in the kit’s Handbook. Then, Relative Quantities (RQ) were calculated as the log base 2 values of the Ct difference between miRNAs and UniSp2 RNA spike-in as described elsewhere (20). Normalisation factor (NF) was calculated as the geometric mean of the RQs of all expressed targets per sample. Normalised Relative Quantities (NRQ) were obtained by dividing the RQs by the sample specific NF.

Data from discovery cohort were normalized using the Global Mean normalization method, as previously described (21). For the miRNAs validation experiments, a group of stable miRNAs was selected from the screening panel using the software packages Normfinder and geNorm. miR-15b-5p, miR-30d-5p, let-7i-5p and miR-15a-5p were chosen to normalise data of the COVID-19 mild vs. severe analysis. Normalisation of CAP and COVID-19 analysis was performed using miR-103a-3p, miR-320a, miR-30e-5p and miR-15b-5p.

### Target Validation by ELISA Arrays

Plasma levels of soluble cytokines and proteins were analysed using a custom ELISA multiplex array (RayBiotech Life, GA, USA) according to manufacturer’s instructions.

### Statistical Analysis

Categorical variables were represented by absolute value and percentage, and continuous variables were represented by median and interquartile range (IQR).

A one-way ANOVA was used to select potential candidate miRNAs with significant differences (p<0.05) across groups (CAP and mild and severe COVID-19 patients) in the screening analysis. Then, of those potential candidates, a multiple linear regression analysis for miRNAs following normal distribution or a logistic regression analysis for miRNAs which could not be transformed to normally-distributed variables were used. Quantitative variables following a non-normal distribution were transformed to normally distributed variables. These models were adjusted by confounding variables such as sex, age, days post-onset of symptoms (POS), ethnic group, or hemolysis. To search for possible confounding variables, Pearson or Spearman correlations were employed. False discovery rate (FDR) correction was assessed by the Benjamini-Hochberg method (22).

Logistic regression analysis for CAP and COVID-19 classification was characterised by using all the variables with a stepwise procedure, with both backward and forward search based on Akaike information criteria, to select the critical variables. The discrimination validity of the score, and also the main value, were assessed by AUC of the ROC curve along with the 95% confidence interval (95%CI).

A Youden’s J statistic was used to determine the sensitivity and specificity of the model. These statistical analyses were performed using our own codes and base functions in R, version 3.5.1 (http://www.R-project.org).

Graphs were performed with GraphPad Prism 8. For outlier detection and removal, ROUT algorithm with an FDR of 1% was used. The rest of the analyses were performed with Stata 14.0 for Windows (Stata Corp LP, College Station, TX).

### Ethics Approval

Transfer of samples from the Biobank and the study protocol was approved by the local Research Ethics Committee (register number 4070) and it was carried out following the ethical principles established in the Declaration of Helsinki. All recruited patients (or their representatives) were informed about the study and gave an oral informed consent as proposed by AEMPS due to COVID-19 emergency.

## Results

### Cohort Selection and Clinical Features of Study Populations

miRNA profile was assessed in a discovery cohort that consisted of 38 COVID-19 patients (20 with mild and 18 with severe disease), collected during the first wave of the pandemic in Spain, from March to April 2020. Median age was 59.5, 15 were female and 23 were male. High blood pressure (HBP) and dyslipidemia (DL) were the most common comorbidities found in the COVID-19 cohort (34.2% and 29% respectively). As a specificity control group, 9 patients (5 female and 4 male) presenting with CAP were included, with a median age of 62.

The validation cohort comprised 85 patients with COVID-19 (43 with mild and 42 with severe disease), recruited during the first wave, and 24 CAP individuals. CAP patients were recruited before pandemic (2014–2015) and in most cases CAP were of unknown etiology. Median age was similar between both cohorts (66.5 and 64), whereas the CAP cohort had higher proportion of males (58.3% versus 41.1% in COVID-19). Among comorbidities, DL (45.9%) and cardiovascular disease (CD) (10.6%) were most frequently found in COVID-19 individuals when compared with CAP (25% and 8.3% respectively). On the other hand, chronic obstructive pulmonary disease (COPD) was higher in CAP patients (20.8% vs 9.4%). The main demographic and clinical characteristics of the study population are described in **Table 1**.

**Table 1:** Demographic and clinical characteristics of the study population.

	Discovery cohort COVID-19			Validation cohort COVID-19			Discovery cohort CAP	Validation cohort CAP
	Total n = 38	Mild n = 20	Severe n = 18	Total n = 85	Mild n = 43	Severe n = 42	n = 9[2] [22.2%]	n= 24
Age	59.5 (51–69)	59 (49.5-69.5)	59.5 (51–38)	64 (55–76)	58 (51–68)	72 (61–83)	62 (59–72)	66.5 (60.5-80)
Sex (male)	23 (60.52)	12 (60)	11 (61.11)	35 (41.18)	13 (30.23)	22 (52.38)	4 (44.44)	14 (58.33)
Days post onset of symptoms	9.5 (7–13)	10 (7.5-14)	8.5 (5–11)	9 (6–12)	9 (7–13)	8 (6–12)	10 (4–15)	6 (3-12.5)
Ethnicity (Caucasian)	26 (68.42)	13 (65)	13 (72.22)	63 (74.1)	31 (72.09)	32 (76.19)	6 (66.67)	22 (95.65)
CURB65	1 (0–2)	1 (0-1.5)	1 (0–2)	1 (0–2)	0 (0–1)	1 (0–2)	1 (1–2)	2.5 (1–4)
Comorbidities	29 (76.32)	15 (75)	14 (77.78)	59 (69.41)	25 (58.14)	34 (80.95)	5 (55.56)	19 (76)
HBP	13 (34.21)	8 (40)	5 (27.78)	36 (42.35)	13 (30.23)	23 (54.76)	2 (22.22)	11 (45.83)
DM	3 (7.89)	0 (0)	3 (16.67)	19 (22.35)	5 (11.63)	14 (33.33)	1 (11.11)	0 (0)
DL	11 (28.95)	7 (35)	4 (22.22)	39 (45.88)	17 (39.53)	22 (52.38)	2 (22.22)	6 (25)
CD	10 (26.32)	5 (25)	5 (27.78)	9 (10.59)	3 (6.98)	6 (14.29)	1 (11.11)	2 (8.33)
COPD	2 (5.26)	0 (0)	2 (11.11)	8 (9.41)	3 (6.98)	5 (11.90)	2 (22.22)	5 (20.83)
Asthma	3 (7.89)	1 (5)	2 (11.11)	2 (2.35)	2 (4.65)	0 (0)	1 (11.11)	1 (4.17)
Immuno -deficiency	4 (10.53)	3 (15)	1 (5.56)	1 (1.18)	0 (0)	1 (2.38)	0 (0)	2 (8.33)
Acute treatment	38 (100)	20 (100)	18 (100)	82 (96.47)	40 (93.02)	42 (100)	7 (77.78)	24 (100)
Lopinavir/ ritonavir	25 (65.78)	11 (55)	14 (82.35)	52 (61.18)	28 (65.12)	24 (57.14)	–	–
Hydroxy-chloroquine	33 (86.84)	18 (90)	15 (88.24)	81 (95.29)	40 (93.02)	41 (97.62)	–	–
Antibiotics*	26 (68.42)	15 (75)	11 (64.71)	69 (81.17)	37 (86.05)	32 (76.19)	7 (77.78)	24 (100)
Beta lactam	–	–	–	–	–	–	3 (33.33)	15 (62.5)
Quinolone	–	–	–	–	–	–	4 (44.44)	3 (12.5)
Macrolide (Clarithromycin/ Azithromycin)	26 (68.42)	15 (75)	11 (64.71)	69 (81.17)	37 (86.05)	32 (76.19)	2 (22.22)	20 (83.33)
Isolated pathogen								
SARS-CoV-2	38 (100)	20 (100)	18 (100)	85 (100)	43 (100)	42 (100)	–	–
Unknown	–	–	–	–	–	–	7 (77.78)	20 (80)
*S. aureus*	–	–	–	–	–	–	1 (11.11)	1 (4)
*S. pneumoniae*	–	–	–	–	–	–	1 (11.11)	3 (12)

All categorical variables are expressed as absolute count (percentage) and quantitative variables as median (Interquartile range). Missing data in each group of patients are expressed as [number] [percentage]. *CAP patients could be treated simultaneously with more than one type of antibiotic.

HBP, high blood pressure; DM, diabetes mellitus; DL, dyslipidemia; CD, cardiovascular disease; COPD, chronic obstructive pulmonary disease; S. aureus, Staphylococcus aureus; S. pneumoniae, Streptococcus pneumoniae.

### Differentially Expressed miRNAs Between COVID-19 and CAP

**Figure 1:**
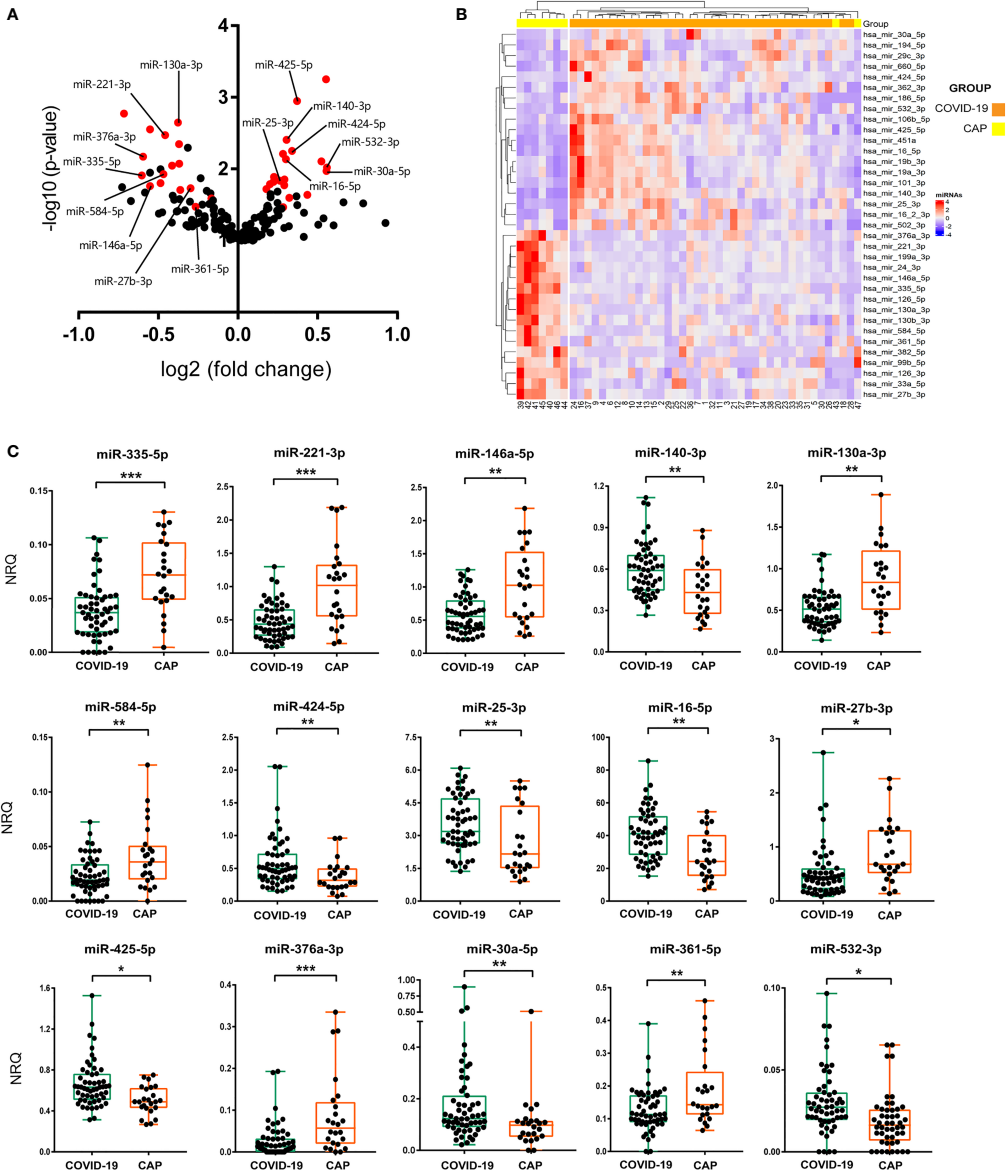
miRNA signature in COVID-19 patients. **(A)** Volcano plot showing differential expression of 179 abundant miRNAs in human plasma between CAP and COVID-19 patients. Log_2_ of fold change of normalised relative quantities (NRQ) and statistical significance (–log_10_ of the p-value) from Mann-Whitney tests for each miRNA were assessed. In red, miRNAs with a corrected p value<0.05 assessed by multivariate statistical analysis. Names of the 15 miRNAs that were further validated are shown. **(B)** Hierarchical clustering heatmap of 35 differentially expressed miRNAs in plasma of 9 CAP and 38 COVID-19 individuals. Red and blue colour indicate upregulated and downregulated expression of COVID-19, respectively, as compared to CAP. **(C)** RT-qPCR of 15 validated miRNAs performed in the validation cohort. Box and whisker plots of NRQ for each miRNA are shown. A group of 4 highly stable miRNAs were used as normalisers. Statistical significance was assessed by means of multivariate statistical tests. *p<0.05, **p<0.01, ***p<0.001.

A screening of 179 miRNAs commonly found in plasma revealed the existence of great differences in the miRNA profile expression between COVID-19 and CAP patients. More than 40 miRNAs showed a significant differential expression (**Figure 1A**). Multiple linear regression analyses were then performed, adjusting by possible confounding variables such as sex, age, hemolysis or POS. Using this approach, we found 35 miRNAs expressed at significantly different levels with a FDR corrected p value<0.05 (**Figure 1B** and **Table 2**). Data analysis revealed 19 upregulated and 16 downregulated miRNAs in COVID-19 vs. CAP patients. To confirm these findings, 21 candidate miRNAs were selected for further validation in an extended cohort of patients (**Supplementary Figure 1**). Candidate miRNAs were chosen based on their expression levels in plasma and their potential biological interest. To confirm the differences detected in the discovery cohort, individual RT-qPCR for every candidate miRNA was carried out in the validation cohort. Multivariate analyses adjusted by POS, age, sex, hemolysis or ethnic group was performed to probe statistical differences between miRNA levels in COVID-19 and CAP. A total of 15 miRNAs displayed differences between groups with a FDR corrected p value<0.05, as shown in **Figure 1C** and **Supplementary Table 1**.

**Table 2:** Differentially expressed miRNAs in COVID-19 vs. CAP patients found in discovery cohort.

miRNA	FDR corrected p-value^e^	Confidence Interval 95%		COVID-19/CAP
**hsa-miR-140-3p**	0.01790	0.5072	1.2578	**↑**
**hsa-miR-335-5p^b^**	0.00895	-0.4236	-0.1462	**↓**
**hsa-miR-27b-3p^a^**	0.00597	-1.1172	-0.3485	**↓**
**hsa-miR-660-5p^a^**	0.00448	0.3304	1.0587	**↑**
**hsa-miR-130a-3p^a^**	0.00358	-1.1136	-0.505	**↓**
**hsa-miR-16-5p^a^**	0.00298	0.5155	1.1372	**↑**
**hsa-miR-146a-5p^a^**	0.00256	-1.7077	-0.7462	**↓**
**hsa-miR-362-3p^b^**	0.00224	0.0898	0.2843	**↑**
**hsa-miR-425-5p^b^**	0.00199	0.2963	0.6154	**↑**
**hsa-miR-101-3p^a^**	0.00179	0.33	0.939	**↑**
**hsa-miR-376a-3p^b^**	0.00163	-0.4307	-0.1448	**↓**
**hsa-miR-126-5p^a^**	0.00149	-1.2407	-0.5048	**↓**
hsa-miR-382-5p**^b^**	0.00138	-0.2923	-0.1191	**↓**
hsa-miR-451a**^b^**	0.00128	7.8489	14.9547	**↑**
hsa-miR-19a-3p**^a^**	0.00119	0.2923	0.8424	**↑**
hsa-miR-19b-3p**^a^**	0.00112	0.2318	0.7337	**↑**
hsa-miR-24-3p**^b^**	0.00105	-1.3138	-0.612	**↓**
hsa-miR-199a-3p**^d^**	0.00099	–	–	**↓**
**hsa-miR-584-5p^b^**	0.00942	-0.3005	0.0837	**↓**
hsa-miR-16-2-3p	0.00895	0.1146	0.3954	**↑**
**hsa-miR-532-3p**	0.01705	0.0476	0.1899	**↑**
**hsa-miR-424-5p^a^**	0.01627	0.2387	1.0115	**↑**
**hsa-miR-25-3p**	0.01557	1.4206	6.1161	**↑**
hsa-miR-99b-5p**^b^**	0.01492	-0.2093	-0.5208	**↓**
**hsa-miR-361-5p^b^**	0.02065	-0.3181	-0.0672	**↓**
**hsa-miR-221-3p^c^**	0.01989	-1.1492	0.8271	**↓**
hsa-miR-194-5p**^b^**	0.03703	0.0689	0.3965	**↑**
**hsa-miR-29c-3p**	0.04042	0.5399	3.2983	**↑**
hsa-miR-33a-5p**^b^**	0.05034	-0.375	-0.0576	**↓**
**hsa-miR-106b-5p^a^**	0.05424	-3.4736	-0.2214	**↑**
hsa-miR-126-3p**^a^**	0.06137	-1.2407	-0.5048	**↓**
hsa-miR-502-3p	0.06289	0.0187	0.148	**↑**
hsa-miR-186-5p	0.07344	0.0166	0.1513	**↑**
hsa-miR-130b-3p**^c^**	0.08055	-4.882	-0.4597	**↓**
**hsa-miR-30a-5p^c^**	0.08098	0.4416	4.8825	**↑**

All miRNAs passing FDR correction (p-value<q-value) are shown. Multiple linear regression analyses were performed except for ^c^(logistic regression) and ^d^(Mann-Whitney tests). The arrows represent up or downregulation for each miRNA in COVID-19 with respect to CAP. miRNAs in bold were selected for validation assays. ^a^log transformed miRNAs, ^b^square root transformed miRNAs, ^c^variable categorisation, ^e^q-value threshold: 0.1.

We next interrogated the possibility that mild and severe COVID-19 patients also displayed specific miRNA signatures. Due to subtle differences found in miRNAs expression between these groups, we chose those miRNAs with an absolute fold change in expression (Ct) higher than 1.3 and a raw p value below 0.1 for further validation (**Supplementary Table 2**). Those miRNAs with a high number of non-detectable values in expression (above 50% of the samples) were not considered. Thereby, we found 10 potential candidate miRNAs (**Supplementary Figure 2**). Only miR-185-5p was downregulated in severe cases of COVID-19. On the other hand, no statistically significant differences in the expression of the candidate miRNAs between COVID-19 mild and severe patients were observed in the validation cohort (**Supplementary Table 3**).

### Logistic Regression Model Based on miRNAs for Classification of COVID-19/CAP Patients

**Figure 2:**
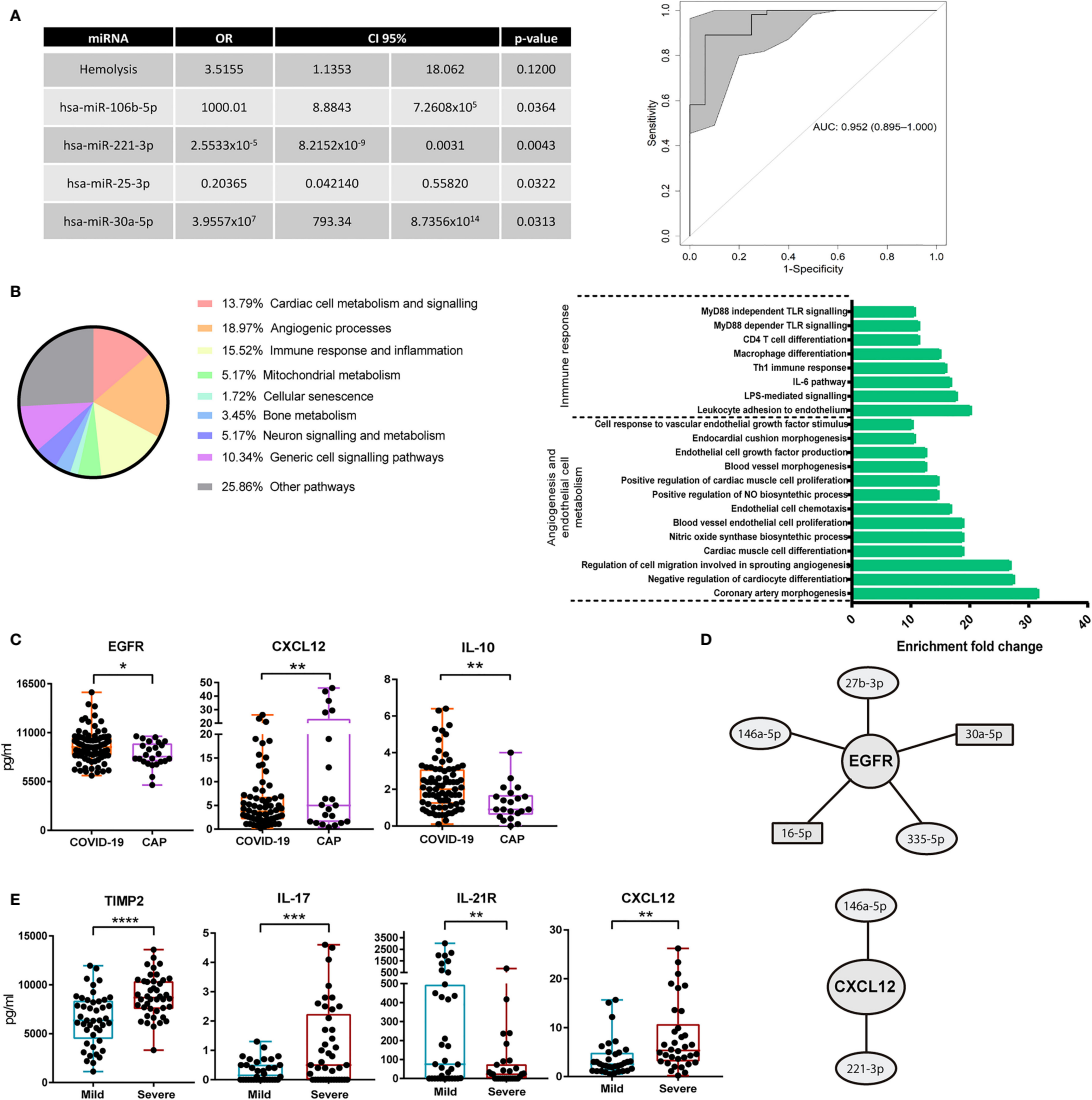
Multivariate regression model for classification of COVID-19 pneumonia and CAP and differences in circulating proteins. **(A)** Left: final logistic regression model for patient classification based on miRNAs. Odds ratio (OR), confidence interval 95% (95%CI) and p-value for each variable were calculated. Stepwise procedure, with both backward and forward search based on Akaike information criteria, to select the critical variables were employed. Right: ROC curve showing the power of discrimination between CAP and COVID-19 of the variables combination shown in the table. Inside the graph, the AUC and its 95%CI. **(B)** Left: relative percentage of each biological process associated with the functional pathways with 10 fold enrichment or higher are shown. Pathway enrichment analysis was assessed using Panther Classification System. Right: enrichment of each individual functional pathway within the two most relevant biological processes. **(C)** Measurement of soluble cytokines in plasma of 24 CAP and 85 COVID-19 individuals. ELISA assays with plasma diluted 1/2 were carried out. Box and whisker plots are shown, statistical significance was assessed by Mann-Whitney tests. *p<0.05, **p<0.01. **(D)** miRNAs regulating EGFR and CXCL12 according to miRTarBase 8.0. Circles show miRNAs with lower expression in COVID-19 as compared to CAP. Rectangles show miRNAs with higher expression in COVID-19 as compared to CAP. **(E)** Box and whisker plots of soluble cytokines in plasma of 43 mild and 42 severe COVID-19 patients. ELISA assays with plasma diluted 1/2 were carried out. Statistical significance was assessed by Mann-Whitney tests. **p<0.01, ***p<0.001, ****p<0.0001.

Next, we developed a multivariate regression model able to differentiate and classify individuals with COVID-19 and CAP. For that purpose, the miRNAs selected for validation assays and other variables including age, sex, ethnic group, POS, IL-6, IgG, IgA, IgM, C3 and C4 were considered. Additional clinical variables shown in **Supplementary Table 4** and main comorbidities: HBP, diabetes mellitus (DM), DL, CD, COPD, asthma and secondary immunodeficiency were also included. Due to the high number of variables tested, a stepwise logistic regression procedure was employed that included the data corresponding to both discovery and validation cohorts. Among all these variables, we only found 4 miRNAs significantly contributing to the regression model: miR-106b-5p, miR-221-3p, miR-25-3p and miR-30a-5p. Hemolysis was included as confounding variable, showing no significant effect on the final model (p=0.12). To assess the performance of this model, a Receiver Operating Characteristic (ROC) analysis was carried out, which displayed a high area under the curve (AUC) value: 0.952 (0.895-1), with a sensitivity of 93.75% and a specificity of 89.09% (**Figure 2A**).

### Analysis of Pathways and miRNA Targets

We next sought to identify the functional profiling of target genes of the 15 validated miRNAs. For that purpose, a Gene Ontology (GO) Enrichment Analysis was performed using Panther Classification System (http://pantherdb.org/). Only those target genes with strong evidence based on functional experiments, according to miRTarBase 8.0 (last accessed 09-22-2020), were considered. Interestingly, most of the enriched pathways were significantly associated with processes related to angiogenesis, regulation of endothelial cells or vasodilatation. Crucial pathways for cardiac muscle cell differentiation and proliferation were also present in the enrichment analysis. Other enriched target genes were related to immune response and inflammatory processes, particularly some controlling leukocyte adhesion to vascular endothelial cells, IL-6 mediated signalling, Th1 response, macrophage differentiation or MyD88 dependent Toll-Like Receptor signalling pathways (**Figure 2B**).

In order to find relevant molecules associated with COVID-19 pneumonia, we measured soluble plasma proteins whose mRNAs were targeted by the identified miRNAs, according to miRTarBase 8.0. We used GO enrichment analysis and Venn diagram tool (http://bioinformatics.psb.ugent.be/webtools/Venn/) to select relevant proteins regulated by several of the validated miRNAs. Thereby, potential targets of our candidate miRNAs associated with angiogenesis pathways (epidermal growth factor receptor, EGFR and vascular endothelial growth factor A, VEGFA), leukocyte adhesion to endothelium (intercellular adhesion molecule 1, ICAM-1), chemokines or cytokines crucial for immune surveillance and lymphocyte proliferation (CXCL12 and IL-21 receptor, IL-21R) or genes involved in cell growth and proliferation (insulin like growth factor receptor 1, IGF1R) were selected for quantification by ELISA. A previous set of cytokine quantification assays performed in a pilot screening of plasma soluble proteins with COVID-19 and CAP patients revealed differences in the plasma levels of IL-11, IL-17 and the tissue inhibitor of metalloproteinases 2 (TIMP-2) (**Supplementary Figure 3**). Hence, these molecules were also quantified in the validation cohort. Finally, IL-10 was also included due to its described relevance in COVID-19 (23).

ELISA assays revealed higher levels of EGFR and IL-10 in COVID-19 vs. CAP patients (**Figure 2C**). Conversely, CXCL12 was downregulated in COVID-19 patients. Both EGFR and CXCL12 were targets of 5 and 2 validated miRNAs, respectively (**Figure 2D**). Likewise, we found differences in the levels of several molecules depending on COVID-19 severity. TIMP-2 and IL-17 were upregulated, while IL-21R was downregulated, in severe cases (**Figure 2E**). The chemokine CXCL12 was also increased in severe vs. mild COVID-19 (**Figure 2E**). Interestingly, 3 out of 8 subjects with higher levels of CXCL12 (above 15 pg/ml) had bacterial superinfection or sepsis.

## Discussion

Growing interest on miRNAs as potential biomarkers or as therapeutic drugs has raised over the last few years, especially in cancer, cardiovascular or neurodegenerative diseases. In this regard, miRNAs may have greater relevance in the future, since they provide us with a better understanding of the pathogenic mechanisms involved in different diseases. Although numerous publications concerning infection by SARS-CoV-2 have been reported in the last year, very few studies addressed its relationship with miRNAs alterations. *In silico* analyses have focused on prediction of miRNAs targeting SARS-CoV-2 genome to find alternative therapies (12, 24). The present study describes a specific miRNA signature in the plasma of COVID-19 patients. A total of 15 miRNAs (7 upregulated and 8 downregulated in COVID-19), with common expression in human plasma and validated in an independent cohort appeared dysregulated in COVID-19 patients compared to CAP patients, revealing important differences in the pathophysiology of these two clinical entities despite their similarities in terms of respiratory symptoms. Due to these differences, we were able to develop a multivariate logistic regression model based on miRNAs that efficiently distinguishes patients with COVID-19 from patients with CAP.

Our study provides experimental evidence that confirms previous *in silico* bioinformatic analyses of possible miRNAs interacting with SARS-CoV-2 genome or playing a role in the host response to the virus. Particularly, dysregulation of miR-424-5p, miR-130a-3p, miR-25-3p, miR-27b-3p and miR-425-5p was predicted in other studies, but it was not verified experimentally (25). Among the miRNAs identified in our study, miR-335-5p was significantly downregulated in COVID-19 vs. CAP patients. This miRNA has been previously associated with suppression of inflammatory processes (26). Its repression during SARS-CoV-2 infection may contribute to the widely described general proinflammatory status (27). In line with these observations, we found low levels of plasma miR-146a-5p in COVID-19 vs. CAP patients. Previous studies have described downregulation of this miRNA during SARS-CoV-2 infection (14). miR-146a regulates inflammation by targeting TNF receptor associated factor 6 (TRAF6), therefore reducing expression of NF-kB (28, 29). Moreover, decreased levels of miR-146a have been linked to higher risk of thrombotic events and neutrophil NETosis (30).

GO enrichment analysis revealed a potential involvement of vascular system biology in this pathology, specifically angiogenesis and response to endothelial damage. This is consistent with several articles that report atherosclerotic plaques, prothrombotic changes in endothelium, increased intussusceptive angiogenesis and a subsequent enhanced risk of thrombosis (31, 32). EGFR is a protein involved in a great number of biological processes; some of them related to blood vessel growth, inflammation *via* NF-kB or profibrotic and atherosclerotic events. In this context, EGFR, a known target of miR-27b-3p, miR-146a-5p, miR-16-5p, miR-335-5p and miR-30a-5p, is found at higher levels in patients with COVID-19, likely enhancing these events (33–35).

Intriguingly, we observed increased levels of the chemokine CXCL12 in CAP vs. COVID-19 patients. CXCL12, which is the CXCR4 ligand, is necessary for effective hematopoiesis, T cell and memory B cell homing to the lymph nodes or monocyte recruitment. Inhibition of this axis is used by several viruses in order to increase their proliferation by reducing the number of circulating immune cells (36). Whether low levels of this chemokine in COVID-19 patients could be triggered by SARS-CoV-2 is a possibility that merits further exploration. miR-146a-5p, miR-221-3p and their target CXCL12 (according to miRTarBase), were downregulated in COVID-19 patients. This observation suggests either non-canonical regulation by these miRNAs or the prevalence of other regulatory mechanisms modulating the expression and secretion of CXCL12. Moreover, CXCL12 was found at higher levels in the plasma of severe vs. mild COVID-19 patients. In our cohort, 3 severe COVID-19 patients, with higher levels of CXCL12, had sepsis or bacterial superinfection. Thus, we cannot rule out the relationship between higher levels of this chemokine and the presence of superinfection or sepsis in critically ill COVID-19 patients, since CXCL12 has a role in neutrophil recruitment from bone marrow (37). The study of the role of miR-221-3p and the interaction with its target, PARP-1, during COVID-19 pathogenesis would be of great interest, since emerging evidence highlights relevant pro and antiviral properties of this protein (38).

IL-17 was found at higher levels in patients with severe COVID-19 disease, in agreement with a prior study indicating its crucial role in the pathogenesis of acute respiratory distress syndrome (ARDS) (39). IL-17 induces recruitment of neutrophils to the lung, exacerbating proinflammatory cytokine release and leading to ARDS. Therefore, the IL-17 signalling pathway could be a potential target to treat the cytokine storm observed in the most severe COVID-19 cases (40). Another well-studied cytokine in the pathogenesis of this disease is IL-10. Several published works show an increase of IL-10 in critically ill COVID-19 patients despite its general anti-inflammatory nature, even proposing this protein as a prognostic biomarker (41, 42), together with other well-known inflammatory cytokines such as IL-6. Here, we report an increased amount of IL-10 in COVID-19 vs. CAP patients. Conversely, no differences were observed between mild and severe disease. Finally, IL-21 is involved in antiviral defence by enhancing Th1 and IFN-γ responses (43). Likewise, its role for a correct B cell memory differentiation and germinal centre reaction leading to effective antibody response could be decisive for viral clearance (44). Thus, low levels of its receptor, IL-21R, in severe COVID-19 could lead to an impaired IFN expression and facilitate the spread and replication of SARS-CoV-2. Receptor proteins like IL-21R or EGFR may be found in plasma by leakage from different cells and tissues. However, caution should be taken when interpreting these results, since soluble EGFR may act as a regulator of its signalling pathway, as previously described (45).

On the other hand, our data revealed clear differences in TIMP-2 levels between severe and mild cases of COVID-19. One of the functions of this inhibitor of matrix metalloproteinases (MMP) is to participate in the regulation of the renewal of the extracellular matrix. Besides this role, it was proposed as an inhibitor of angiogenesis by MMP-independent mechanisms (46). Therefore, this protein may be participating in the response to direct endothelial damage caused by SARS-CoV-2.

One of the limitations of this study is the low number of patients with mild disease, which preclude to identify differences in miRNAs between mild and severe COVID-19, as most of the studied subjects belong to moderate and severe subgroups of patients. Furthermore, sample collection days vary between COVID-19 (within first 5 days upon admission) and CAP (day right after the admission). However, no significant difference in days post-symptoms onset between CAP and COVID-19 in discovery cohort (p=0.98) and validation cohort (p=0.07) were found. Another limitation is the variety of treatments within COVID-19 and CAP patients, which makes hard the extrapolation of these results. On the other hand, the main challenge when using RT-qPCR for miRNA expression analysis and its implementation in clinical practice is the lack of a standard method of normalisation among different laboratories. Further studies on miRNAs derived from lung and other tissues are needed to delve into our knowledge of the pathogenesis of this disease.

Despite these limitations, this study conclusively proves the existence of a differential profile in circulating miRNAs between COVID-19 and CAP patients. Analysis of the identified miRNAs showed regulatory functions associated with angiogenesis and inflammation, indicating that endothelial damage and vascular compromise is definitively one of the main conditions driven by SARS-CoV-2. Here, we describe new miRNAs and soluble cytokines that may contribute to gain insight into COVID-19 pathogenic mechanisms and set the basis for the urgently needed design of novel therapeutic strategies.

## Data Availability Statement

The original contributions presented in the study are included in the article/**Supplementary Material**. Further inquiries can be directed to the corresponding author.

## Ethics Statement

The studies involving human participants were reviewed and approved by Hospital Universitario de la Princesa. The patients/participants provided their written informed consent to participate in this study.

## Author Contributions

Conception and design: PM-F, PV-T, MJ-F, SR, CL-S, ML-P, HF, and FS-M. Acquisition of data and samples: PM-F, PV-T, ER-V, AV, AL-S, JMG-R, and JA. Statistical analyses: PM-F, PV-T, MJ-F, SR, and AS-G. Drafting the manuscript: PM-F and PV-T. Revising the manuscript: PM-F, PV-T, MJ-F, SR, ML-P, ER-V, AS-G, IG-Á, AA, CM-C, JMG-R, JA, IG-A, AA , FS-M, and EM-G. Obtaining financial support: IG-A, AA, and FS-M. All authors contributed to the article and approved the submitted version.

## Funding

This study was funded by Spanish Ministry of Economy, Industry and Competitiveness (MINECO) and Instituto de Salud Carlos III (grant nos. RD16/0011/0012 and PI18/0371 to IG-Á, grant no. PI19/00549 to AA, and grant no. PDI-2020-120412RB-I00 to FSM) and co-funded by the European Regional Development Fund. The study was also funded by ”La Caixa Banking Foundation” (grant no. HR17-00016 to FSM), REACT-UE INMUNOVACTER-CM from Comunidad de Madrid, and ”Fondos Supera COVID19” by Banco de Santander and CRUE. The work of ER-V has been funded by a Rio-Hortega grant from the Ministerio de Economía y Competitividad (grant no. CM19/00149 Instituto de Salud Carlos III) and co-funded by The European Regional Development Fund (ERDF) “A way to make Europe”.

## Conflict of Interest

The authors declare that the research was conducted in the absence of any commercial or financial relationships that could be construed as a potential conflict of interest.

## Publisher’s Note

All claims expressed in this article are solely those of the authors and do not necessarily represent those of their affiliated organizations, or those of the publisher, the editors and the reviewers. Any product that may be evaluated in this article, or claim that may be made by its manufacturer, is not guaranteed or endorsed by the publisher.
