# Supplementary material for: A Differential Signature of Circulating miRNAs and Cytokines Between COVID-19 and Community-Acquired Pneumonia Uncovers Novel Physiopathological Mechanisms of COVID-19
Source: Front Immunol. 2022 Jan 11;12:815651. doi: 10.3389/fimmu.2021.815651 (PMC8787267; doi:10.3389/fimmu.2021.815651)
Supplement: Supplementary file 1 [file DataSheet_1.pdf]

Supplementary Material

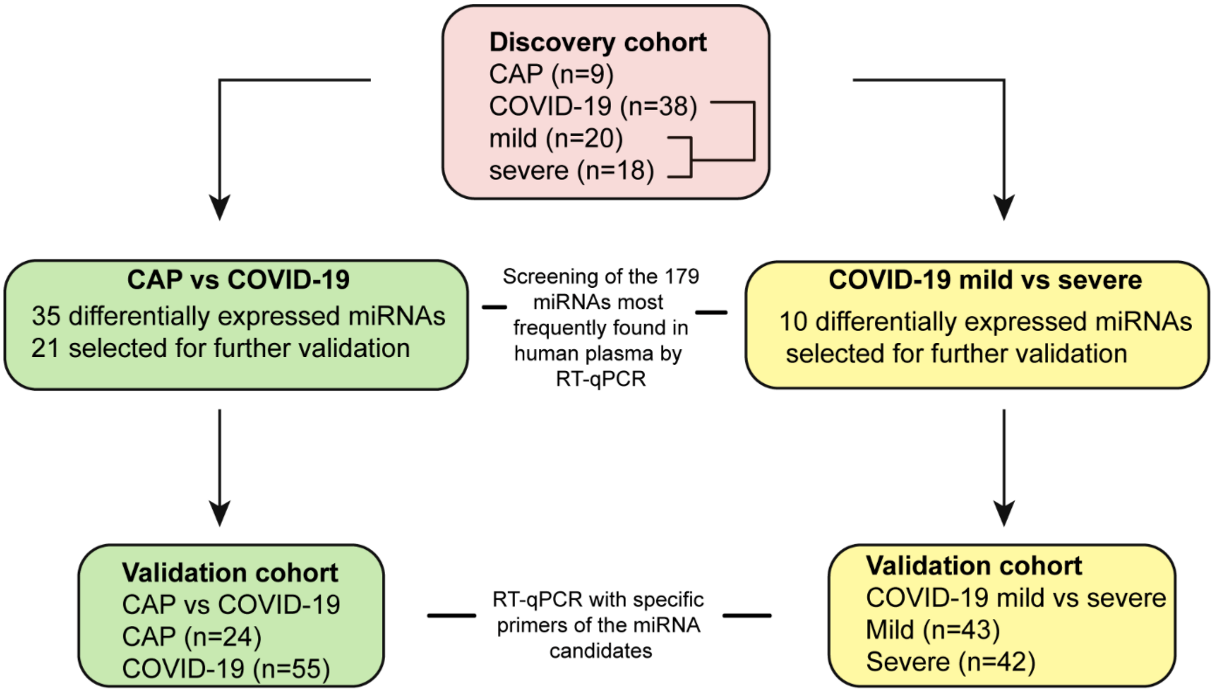

Supplementary Figure 1. Diagram of the experimental design. Composition of the discovery cohort, where a first screening of miRNAs was carried out, and composition of the extended cohort to validate the preliminary results.

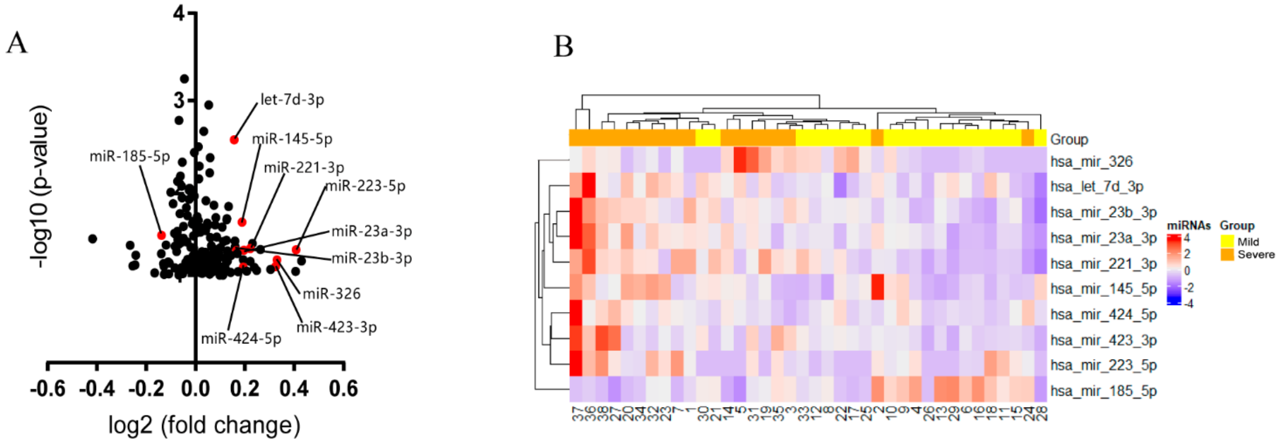

Supplementary Figure 2. Differential expression of miRNAs in mild and severe COVID-19. **A)** Volcano plot showing differential expression of 179 abundant miRNAs in human plasma between COVID-19 mild and severe patients. In red, miRNAs with an absolute fold change higher than 1.3 and a raw p value below 0.1. **B)** Hierarchical clustering heatmap of 10 differentially expressed miRNAs in plasma of 20 mild and 18 severe COVID-19 individuals.

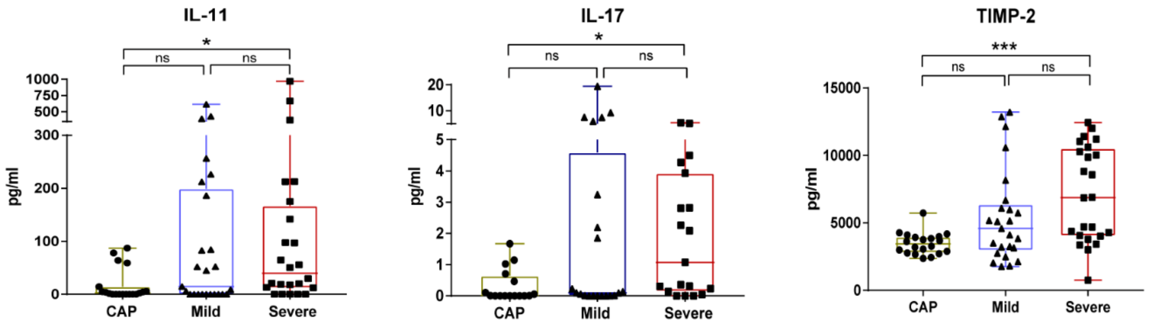

Supplementary Figure 3. Screening of plasma circulating proteins. Box and whisker plots showing levels of soluble proteins in plasma of 20 CAP and 50 COVID-19 individuals (25 mild and 25 severe). ELISA assays with plasma diluted 1/2 were carried out. Statistical significance was assessed by Kruskal-Wallis tests. *p<0.05, ***p<0.001.

miRNA	FDR corrected p-value[e]	Confidence Interval 95%		COVID-19/CAP
**hsa-miR-335-5p**	0.0021	-0.0478	-0.0198	↓
**hsa-miR-221-3p[a]**	0.0021	-0.9089	-0.2687	↓
**hsa-miR-376a-3p[d]**	0.0021	-	-	↓
**hsa-miR-146a-5p[b]**	0.0021	-0.3035	-0.0876	↓
**hsa-miR-140-3p**	0.0210	0.0546	0.2416	↑
**hsa-miR-130a-3p[a]**	0.0140	-0.6306.	-0.1479	↓
**hsa-miR-584-5p[b]**	0.0158	-0.0867	-0.0177	↓
**hsa-miR-424-5p[a]**	0.0168	0.1476	0.7399	↑
**hsa-miR-25-3p[a]**	0.0245	0.0851	0.5251	↑
**hsa-miR-30a-5p[c]**	0.0210	0.4403	2.7693	↑
**hsa-miR-361-5p[d]**	0.0236	-	-	↓
**hsa-miR-16-5p**	0.0210	2.4966	17.0983	↑
**hsa-miR-532-3p[c]**	0.0210	0.3390	2.4687	↑
**hsa-miR-27b-3p[a]**	0.0325	-0.8434.	-0.0850	↓
**hsa-miR-425-5p[a]**	0.0438	0.0235	0.3432	↑
hsa-miR-106b-5p[b]	0.0662	0.0034	0.1596	-
hsa-miR-101-3p[b]	0.0900	-0.0050	0.2454	-
hsa-miR-126-5p[a]	0.1694	-0.4439.	0.0529	-
hsa-miR-29c-3p[d]	0.3688	-	-	-
hsa-miR-362-3p[d]	0.7881	-	-	-
hsa-miR-660-5p[b]	0.9520	-0.0485.	0.0613	-

Supplementary Table 1. Validation of differentially expressed miRNAs in COVID-19 vs. CAP. Bold: miRNAs passing FDR (p-value<q-value). Multiple linear regression analyses were performed except for [c] (logistic regression) and [d] (Mann-Whitney tests). [a] log transformed miRNAs, [b] square root transformed miRNAs, [c] variable categorisation. [e] q-value threshold: 0.05. The arrows represent up or downregulation for each miRNA in COVID-19 with respect to CAP.

miRNA	Fold change (NRQ)	p-value
hsa-miR-23a-3p	1.567	0.0008
hsa-miR-221-3p	1.664	0.0019
hsa-miR-223-5p	2.554	0.0094
hsa-miR-23b-3p	1.460	0.0179
hsa-miR-185-5p	0.726	0.0244
hsa-miR-145-5p	1.543	0.0305
hsa-miR-424-5p	1.557	0.0328
hsa-let-7d-3p	1.432	0.0437
hsa-miR-423-3p	2.107	0.0573
hsa-miR-326	2.135	0.0643

Supplementary Table 2. Differentially expressed miRNAs in COVID-19 mild and severe in discovery cohort. Differences of normalised relative quantities (NRQ) in severe vs. mild cases expressed in fold change. Mann-Whitney individual tests for each miRNA were performed to assess statistical significance (p-value shown). Table shows those miRNAs selected for validation assays, with an absolute fold change above 1.3 and a p-value below 0.1.

miRNA ID	FDR corrected p-value[d]	Confidence Interval 95%	
hsa-let-7d-3p[c]	1.050	-0.1657	1.7595
hsa-miR-424-5p[a]	0.615	-0.0563	0.4655
hsa-miR-423-3p[a]	0.413	-0.0624	0.5058
hsa-miR-145-5p[c]	0.403	-1.0699	0.2664
hsa-miR-223-5p	0.338	-0.0022	0.0124
hsa-miR-23a-3p[a]	0.603	-0.1018	0.2758
hsa-miR-326[c]	0.534	-1.3619	0.5124
hsa-miR-23b-3p[a]	1.018	-0.2057	0.261
hsa-miR-185-5p	0.939	-0.2208	0.1811
hsa-miR-221-3p[b]	0.944	-0.081	0.0755

Supplementary Table 3. Statistical analysis of miRNAs in mild vs. severe COVID-19 in the validation cohort. Multiple linear regression analyses were performed except for [c] (logistic regression). [a] log transformed miRNAs, [b] square root transformed miRNAs, [c] variable categorisation. [d] q-value threshold: 0.1

	Discovery cohort COVID-19			Validation cohort COVID-19			Discovery cohort CAP	Validation cohort CAP
	Total n = 38	Mild n = 20	Severe n = 18	Total n = 85	Mild n = 43	Severe n = 42	n= 9	n= 24
Platelets (10^3/mm^3)	189 (140-276)	214.5 (139-344)	184 (149-229)	234 (184-278)	236.5 (189-278)	234 (184-276)	304 (198-457)	204 (169-290)
Leukocytes (10^3/mm^3)	7.15 (5.44-8.56)	5.86 (2.91-8.06)	8.20 (7.00-9.22)	5.42 (4.49-7.53)	4.99 (4.05-6.25)	5.89 (4.83-8.50)	8.7 (4.96-12.77)	8.9 (7.08-11.11)
Lymphocytes (10^3/mm^3)	1.23 (0.74-1.63)	1.22 (0.83-1.90)	1.23 (0.67-1.43)	1.31 (0.89-1.76)	1.43 (1.06-1.77)	1.05 (0.85-1.66)	1.29 (0.86-1.4)	1.05 (0.63-1.62)
Monocytes (10^3/mm^3)	0.47 (0.31-0.65)	0.49 (0.27-0.72)	0.46 (0.34-0.58)	0.47 (0.30-0.64)	0.41 (0.30-0.64)	0.47 (0.30-0.57)	-	-
Eosinophils (10^3/mm^3)	0.02 (0.00-0.08)	0.025 (0.01-0.09)	0.02 (0.00-0.07)	0.03 (0.01-0.11)	0.025 (0.01-0.13)	0.03 (0.00-0.08)	-	-
Creatinine (mg/dL)	0.85 (0.72-0.99)	0.775 (0.7-0.885)	0.95 (0.75-1.09)	0.75 (0.64-0.96)	0.72 (0.67-0.85)	0.83 (0.64-1)	0.66 (0.61-0.81)	0.875 (0.605-1.115)
K+ (mEq/L)	4.1 (3.7-4.4)	4.1 (3.7-4.4)	4.15 (3.7-4.4)	4.2 (3.8-4.5)	4.2 (3.7-4.5)	4.3 (3.85-4.65)	4.21 (3.87-4.51)	4.125 (3.78-4.22)
Bilirubin (mg/dL)	0.46 (0.37-0.71)	0.46 (0.37-0.68)	0.465 (0.34-0.76)	0.46 (0.32-0.615)	0.42 (0.3-0.62)	0.46 (0.36-0.61)	0.35 (0.25-1.08)	0.37 (0.3-0.595)
GOT (U/L)	30.5 (22-50)	27 (17.5-49)	33 (25-51)	26 (21-41)	23 (17-36)	30 (23-44)	43 (26-81)	20.5 (16-41)
GGT (U/L)	54.5 (36-117)	49.5 (20-117)	55 (38.5-121.5)	39 (23.5-61.5)	33.5 (17-51)	45 (28-100)	-	-
GPT (U/L)	33 (20-53)	31 (18.5-51.5)	33 (20-79)	26.5 (17.5-51)	22 (17-42)	31 (19-56)	61 (30-74)	16 (12-35)
LDH (U/L)	241.5 (206-300)	219 (201-252)	294 (228-474)	236.5 (207-293)	225 (184-260)	279 (225-313)	233 (179-260)	180 (158-232)
Ferritin (ng/mL)	948 (493-1624)	582 (370-1293)	1344 (894-1878)	544 (217-805)	384.5 (187-642)	645 (456-1324)	-	-
CRP (mg/dL)	5.77 (2.55-12.95)	4.22 (2.17-9.93)	9.26 (3.54-25.18)	4.14 (2.09-8.95)	2.67 (1.07-5.52)	6.47 (3.95-13.81)	8.4 (2.2-22.8)	9.55 (5-14.8)
Prothrombin time (sec)	12.5 (11.75-13.15)	12.5 (12.1-13.1)	12.1 (11.7-13.5)	11.9 (11.4-12.65)	11.95 (11.4-12.5)	11.85 (11.3-12.7)	-	-
INR	1.14 (1.07-1.21)	1.15 (1.08-1.21)	1.11 (1.07-1.22)	1.09 (1.05-1.15)	1.09 (1.05-1.13)	1.095 (1.04-1.18)	1.03 (0.97-1.19)	1.04 (0.98-1.15)

Supplementary Table 4. Clinical variables of the study population. All variables are expressed as median (Interquartile range). *K+, potassium; GOT, glutamic oxaloacetic transaminase; GGT, gamma-glutamyl transferase; GPT, glutamate pyruvate transaminase; LDH, lactate dehydrogenase; CRP, C-reactive protein; INR, international normalised ratio.*
